# Effects of chemical additive and packing density on the fermentation profile and nutrient composition of ensiled cocktail forage mix

**DOI:** 10.3168/jdsc.2022-0350

**Published:** 2023-04-20

**Authors:** M.S. Souza, M.R. Pupo, E.C. Diepersloot, M.S. Akins, L.F. Ferraretto

**Affiliations:** 1 Department of Animal and Dairy Sciences, University of Wisconsin–Madison 53718; 2 Department of Animal Science, Federal University of Amazônia, Belém, PA, Brazil 66077-830; 3 USDA-Agricultural Research Service, US Dairy Forage Research Center, Marshfield, WI 54449

## Abstract

Recently, the use of cocktail forage mixes in dairy cattle rations has become more common because the mixtures are low-cost, fit well in rotation after a cereal grain forage, and can have similar yield and energy value compared with alfalfa silage. This experiment evaluated the effects of a chemical additive and packing density on the fermentation profile and nutrient composition of cocktail mix silage. The cocktail forage mix (brown-midrib sorghum-sudangrass, Italian ryegrass, red clover, berseem clover, and hairy vetch) was harvested, ensiled in laboratory silos (3.79-L plastic buckets), and allowed to ferment for 30 d. The experiment consisted of 6 treatments, 2 chemical additives [CON (30 mL of distilled water) or ADD (sodium sulfite, sodium metabisulfite, and fungal amylase)], and 3 packing densities [D100, D75, and D50 (100%, 75%, or 50% of the maximum material in laboratory silos, respectively)], for a total of 24 silos (4 replications per treatment combination). No interactions of additive by density were detected for any parameters evaluated. The addition of the chemical additive influenced fermentation profile, with reduced concentrations of total acids, lactic acid, acetic acid, and ethanol in ADD-treated silages. Moreover, D50 reduced concentrations of total acids, lactic acid, and acetic acid compared with D100, but had greater pH and yeast and mold counts. Minimal changes in nutrient composition were detected regardless of treatment. Overall, this study corroborates the importance of a well-packed silage during the ensiling process. Poorly packed cocktail mix silages may be more prone to spoilage based on yeast and mold counts.

## Highlights

• No interactions between chemical additive and density were observed.
• Chemical additive decreased total acid production and increased pH.
• Greater density increased total acid production and decreased pH.
• No treatments affected nutrient composition of cocktail mix silage.

## Graphical Abstract

**Figure:**
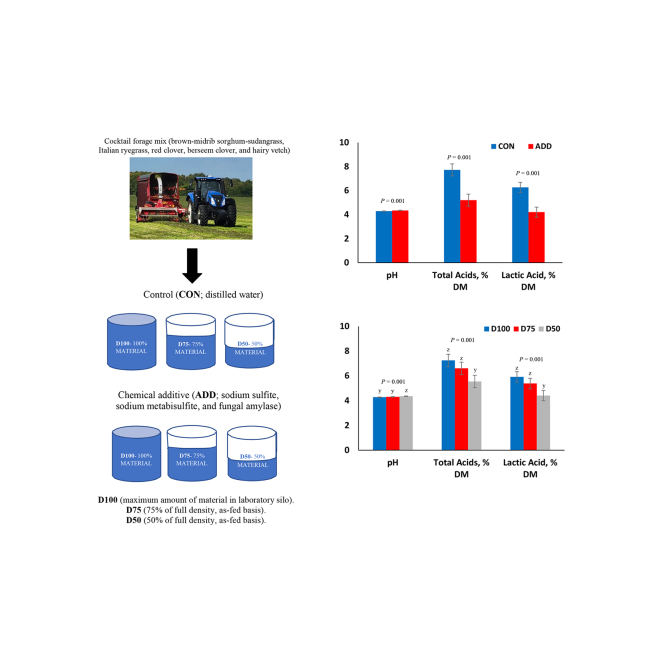
**Summary:** The objective of this experiment was to evaluate the effects of a chemical additive and packing density on the fermentation profile and nutrient composition of a cocktail mix silage. Forage subsamples were collected, and each experimental unit was individually treated without (CON; 30 mL of distilled water) or with a sodium sulfite, sodium metabisulfite, and fungal amylase additive solution (ADD; containing a minimum of 8% sulfur and targeting for 0.11% of fresh forage according to manufacturer recommendation), and hand-mixed with harvested forage immediately before ensiling. Forage was ensiled in laboratory silos (3.79-L plastic buckets) with maximum packing density (D100, 215 kg of DM/m^3^), 75% of the maximum density (D75, 161 kg of DM/m^3^), or 50% of the maximum density (D50, 108 kg of DM/m^3^). Even though fermentation was adequate for all silage samples, poorly packed cocktail forage mix silages had greater yeast and mold counts and a less pronounced fermentation.

Cocktail forage mixes are often used as cover crops to provide physical, chemical, and biological benefits to the soil (Fageria et al., 2005). However, selecting species within a mixture for forage quality could offer additional benefits by supporting the requirements of dairy cattle through a low-cost forage source. Although forage mixes containing cool season crops seeded later in the growing season may yield forage with high nutritive value (Neville et al., 2008), successful production of silage can be challenging for dairy producers because some legumes and grasses included in these cocktail forage mixes (i.e., ryegrass or clover) have a greater buffering capacity compared with other crops commonly grown for dairy cattle (Harrison et al., 2003). High buffering capacity [i.e., high ammonia or cation mineral (Ca, K, Mg) concentrations, or both] can make silage more susceptible to DM and nutrient losses, undesirable fermentation (particularly clostridial or butyric acid–based fermentation), and promotes aerobic spoilage in the ensiled material (Wilkinson and Davies, 2013; Kung et al., 2018).

Packing density is important to maintain silage nutritive value by avoiding air exposure, which increases aerobic respiration and heat production (Muck et al., 2004). Greater porosity delays anaerobic fermentation, allowing the growth of aerobic microorganisms that consume readily available carbohydrates and organic acids (Pitt and Muck, 1993). Ruppel et al. (1995) found that the packing density was negatively correlated with storage losses, which increased up to 20 percentage units when packing density decreased from 320 to 160 kg of DM/m^3^. Poorly packed silage may benefit from the use of chemical additives, reducing nutrient losses from the silo and improving silage palatability. Sodium metabisulfite has strong reducing capacity, consuming large amounts of O_2_, and stimulating rapid anaerobic conditions in the silos (Kung et al., 2003). In addition, sulfur dioxide, a product of the reaction between sulfur metabisulfite and moisture or acids, is a powerful antimicrobial agent (Kung et al., 2003). McDonald et al. (1991) reported that silages treated with sodium metabisulfite had greater pH, lower concentration of fermentation acids, reduced proteolysis and deamination, and decreased DM and nutrient losses compared with the corresponding untreated silages. Likewise, Ahmadi et al. (2021) observed that sodium metabisulfite can reduce fermentation, decreasing total acid production in silage, and the growth of undesirable microorganisms, such as yeast and molds. To our knowledge, studies evaluating the effects of packing density and sulfur-based additives in cocktail mixed forages are lacking in the literature.

Thus, the objective of this experiment was to evaluate the effects of a chemical additive and packing density on the fermentation profile and nutrient composition of cocktail mix silage. We hypothesized that the chemical additive would improve fermentation profile of cocktail mix silage, but this response would be greater when silage was poorly packed.

This experiment was carried out in a completely randomized block design with a 2 × 3 factorial arrangement of treatments and consisted of 6 treatments (2 chemical additives × 3 packing densities) with 4 replications (order of forage load emptying considered a block) for a total of 24 laboratory silos.

Cocktail mix forage was seeded in a field of the University of Wisconsin Marshfield Agricultural Research Station (Marshfield, WI) with 22, 10, 5, 5, and 5 kg/ha of brown-midrib sorghum-sudangrass (Nutri-King BMR 6, Gayland Ward Seed), Italian ryegrass (Kingfisher Allegro mix, Byron Seeds), red clover (Marathon, Welter Seed and Honey Co.), berseem clover (Frosty, GO Seed), and hairy vetch (AU Merit, Smith Seed Services), respectively. Second-cut cocktail forage mix was mowed on September 15, 2021, using a MacDon Model R113 pull-type mower/conditioner (MacDon Industries Ltd.), to a 10-cm stubble height. This mower-conditioner is configured with a maximum 4.0-m cutting width, and forage is subsequently conditioned with 3.3-m-wide steel intermeshing conditioning rollers. Forage was allowed to wilt (18 h) to approximately 39% DM, and 2 windrows merged immediately before harvest on September 16, 2021, at 1000 h using a pull-type forage harvester (model F-41, Dion-AG) set for a theoretical length of cut of 1.27 cm. Forage was chopped directly into a forage wagon and immediately transported to the laboratory. No inoculant or other additive was added to the forage during harvest. Forage subsamples were collected during unloading of forage from the forage wagon (1 sample/block) and analyzed for pH, microbial counts, and nutrient characterization of fresh, unfermented material.

Chopped forage was unloaded from the forage wagon in amounts sufficient for each block of silos to be made at a time to minimize drying effects on the forage. Forage subsamples (2.04, 1.59, and 1.14 kg for maximum density, 75% of maximum density, and 50% of the maximum density, on average, respectively) from each unloading time were collected and each laboratory silo was individually treated without (**CON**; 30 mL of distilled water) or with a sodium sulfite, sodium metabisulfite, and fungal amylase additive solution (**ADD**; containing a minimum of 8% sulfur and targeting for 0.11% of fresh forage according to manufacturer recommendation), and hand-mixed with harvested forage immediately before ensiling. The amount of ADD was adjusted depending on the forage amount used in each packing density treatment. The chemical additive contained a combination of sodium sulfite, sodium metabisulfite, and fungal amylase.

Forage was ensiled in laboratory silos (3.79-L plastic buckets) with maximum packing density (**D100**, 215 kg of DM/m^3^), 75% of the maximum density (**D75**, 161 kg of DM/m^3^) or 50% of the maximum density (**D50**, 108 kg of DM/m^3^). All silos were sealed with a lid and tape, weighed, and then stored at room temperature (22.3 ± 0.3°C) until reaching 30 d of storage length. After reaching 30 d of fermentation, laboratory silos were weighed, opened, and the top 4 cm of material was discarded. Subsamples were collected for pH, microbial counts, fermentation profile, and nutrient characterization. Dry matter losses were calculated as the difference between the weight of the material placed in each laboratory silo at ensiling and the weight of the silage after the designated length of storage divided by the weight of material at ensiling, corrected for the respective initial or final DM concentration (Jobim et al., 2007). Laboratory silos containing approximately 350 g of remaining silage that had been mixed and loosely replaced after sample collections were used for determination of aerobic stability. Wireless temperature sensors (HOBO temperature data logger 64 k; Onset Computer Corporation) set to record temperatures every 30 min for 240 h were placed in the geometric center of laboratory silos. Three additional sensors were also placed to record ambient temperature, which averaged 22.3 ± 0.3°C for the duration of the aerobic stability assay. Aerobic stability was defined as the number of hours before silage increased 2°C above the baseline of room temperature.

Extracts of fresh and silage samples were obtained from 20 g of an undried, unground sample diluted 10-fold (mass basis) in 0.1% peptone water (Oxoid CM0090), blended for 60 s in a high-speed blender, and filtered through 2 layers of cheese cloth. The extract was divided into 2 subsamples. The first subsample was used to measure pH in duplicate using a pH electrode (Accumet AP85 Portable Waterproof; Thermo Fisher Scientific Inc.). The second subsample was evaluated for yeast and mold enumeration via a pour plating method in a 10-fold serial dilution on malt extract agar (Difco 211220) acidified with 85% lactic acid. Agar plates were incubated at 32°C for 48 h for yeast, and an additional 72 h for mold counts. Yeast and mold counts were evaluated on the same plates and separated by visually examining the growth of colonies.

Silage samples were submitted to a commercial laboratory (Dairyland Laboratories Inc., Arcadia, WI) for fermentation profile analysis. Organic acids (lactic, acetic, propionic, and butyric acids), ethanol, and 1,2-propanediol were analyzed by HPLC (Canale et al., 1984). Ammonia nitrogen was determined by colorimetry using a Technicon Automatic Analyzer (RFA-300, Alpkem Corporation) with a method adapted from Noel and Hambleton (1976).

Fresh and silage samples were dried in a forced-air oven set at 60°C for 48 h to determine DM concentration, ground to pass through a 1-mm sieve using a Wiley Mill (Thomas Scientific), and sent to a commercial laboratory (Dairyland Laboratories Inc.) for nutrient characterization. Dried, ground samples were analyzed by near-infrared spectroscopy (Foss model 5000; Foss North America Inc.) with calibration equations for ash, water-soluble carbohydrates, ether extract, CP, neutral detergent fiber treated with α-amylase and sodium sulfite (aNDF), neutral detergent fiber treated with α-amylase and sodium sulfite, corrected for residual ash (aNDFom), and starch based on wet chemistry procedures described in AOAC method 942.05 (AOAC International, 2005), Derias (1961), AOAC method 920.39 (AOAC International, 2005), AOAC method 990.03 (AOAC International, 2005), AOAC method 2002.04 (AOAC International, 2005), AOAC method 2002.04 (AOAC International, 2005), and Vidal et al. (2009), respectively. The digestibility of NDF after 30 h was determined using a method adapted from Goering and Van Soest (1970), and residue was analyzed for aNDFom concentration by near-infrared spectroscopy as described before.

Fresh samples were not analyzed statistically; these samples were only used to characterize nutrient composition of forage before treatment and ensiling (Table 1). Data from silage samples were analyzed as a completely randomized block design in a 2 × 3 factorial arrangement of treatments using general linear mixed-model procedures (PROC GLIMMIX, SAS 9.4; SAS Institute Inc.) with a model including the fixed effects of chemical additive, packing density, and their interaction. Forage sampling order (block) was the sole random effect. Means were determined using the LSMEANS statement. Significance was declared at *P* ≤ 0.05.

**Table 1:** Nutrient composition, pH, and yeast and mold counts of harvested, fresh cocktail forage mix (average ± SD)

Item^1^	Forage cocktail mix
pH	6.1 ± 0.04
DM, % as fed	39.1 ± 3.47
CP, % of DM	11.6 ± 0.25
EE, % of DM	3.6 ± 0.02
NDF, % of DM	53.1 ± 0.52
Starch, % of DM	3.2 ± 0.31
Ash, % of DM	8.1 ± 0.21
WSC, % of DM	10.7 ± 0.14
Yeast counts, log cfu/g	3.9 ± 0.25
Mold counts, log cfu/g	3.8 ± 0.10

1 EE = ether extract; WSC = water-soluble carbohydrates.

No interaction effects were observed (*P* > 0.05) in any of the parameters evaluated. Therefore, main effects are presented and discussed in the absence of a significant 2-way interaction. Propionic acid, butyric acid, and 1,2-propanediol were measured but below detection limits for all samples. Furthermore, all samples were stable for 240 h, the maximum amount of time silos remained under evaluation for aerobic stability, regardless of treatment. Even though literature is conflicting about the effects of sodium metabisulfite on aerobic stability (Kung et al., 2003), packing density is well known to change aerobic stability. In the present study, however, the small amount of cocktail mix used to measure aerobic stability was possibly insufficient to detect any difference as the heat generated was likely dissipate rapidly from the silage mass. Further research is warranted to investigate aerobic stability in cocktail mix silage.

Effects of chemical additive and packing density on fermentation profile and microbial counts are shown in Table 2. Silage treated with ADD had lower (*P* ≤ 0.01) concentrations of total acids, lactic acid, acetic acid, and ethanol compared with CON. However, the pH of ADD silage was greater (*P* = 0.001) than CON silage. Chemical additives containing sodium sulfite and sodium metabisulfite are readily available and effective preservatives with known bactericidal properties. Similar products are widely used in the food industry for preservation by suppressing microbial growth (Natskoulis et al., 2018). These additives are also used to influence fermentation in silage to restrict cellular respiration in the ensiled material. Cellular respiration is restricted by the liberation of sulfur dioxide (SO_2_), which contributes to the formation of an anaerobic environment (Knodt, 1950). Previous studies reported sodium sulfite and sodium metabisulfite can suppress microorganisms involved in protein degradation and ammonia formation (Gould and Russell, 2003; Ahmadi et al., 2018). Also, literature evaluating sodium metabisulfite as a preservative for legume-grass silage observed a reduction in organic acids and ammonia, increasing silage pH (Mathison et al., 1979). Similarly, Romero-Gil et al. (2016) found that sodium metabisulfite was an efficient additive to reduce the growth of lactic acid bacteria and yeast. Conversely, no effects of chemical additive on yeast and mold counts were observed in this experiment.

**Table 2:** Effects of chemical additive and packing density on the fermentation profile and microbial counts of ensiled cocktail forage mix^1^

Item	CON			ADD			SEM^2^	*P*-value		
	Full	75%	50%	Full	75%	50%		AD	DEN	AD × DEN
pH	4.26	4.28	4.32	4.31	4.34	4.40	0.02	0.001	0.001	0.64
Total acids, % of DM	8.2	8.2	6.8	6.3	5.0	4.3	0.50	0.001	0.001	0.17
Lactic acid, % of DM	6.6	6.7	5.5	5.2	4.1	3.3	0.42	0.001	0.001	0.16
Acetic acid, % of DM	1.5	1.5	1.4	1.1	0.9	0.9	0.09	0.001	0.03	0.25
Ethanol, % of DM	0.5	0.6	0.6	0.5	0.3	0.5	0.08	0.01	0.31	0.08
1,2 Propanediol, % of DM	0.06	0.09	0.12	0.02	0.01	0.07	0.02	0.001	0.01	0.54
Ammonia-N, % of CP	3.7	3.8	3.7	3.7	3.7	3.6	0.22	0.62	0.60	0.73
Yeast, log cfu/g	3.55	4.60	5.36	4.25	4.74	6.10	0.71	0.21	0.01	0.80
Mold, log cfu/g	3.88	3.39	4.57	3.61	3.52	5.28	0.71	0.64	0.02	0.62

1 Treatments consisted of AD: effect of additive (CON: 30 mL of distilled water; ADD: sodium sulfite, sodium metabisulfite, and fungal amylase; 0.11% of fresh forage); DEN: effect of packing density [D100 (maximum amount of material in laboratory silo), D75 (75% of full density, as-fed basis), and D50 (50% of full density, as-fed basis)].

2 Greatest SEM.

Cocktail mix silage ensiled at D50 had lower (*P* ≤ 0.03) concentrations of total acids, and lactic and acetic acids compared with D100. Zhang and Yu (2015) evaluated the effects of 2 different levels of packing density (500 vs. 600 kg as fed/m^3^) on the fermentation profile of grass silage and observed a greater lactic acid concentration, coupled with a lower pH, butyric acid concentration, and ammonia-N concentration in silage with the greater density. Likewise, Savage et al. (2015) observed greater lactic acid concentration in corn silage ensiled at 240 kg of DM/m^3^ compared with 170 kg of DM/m^3^. In the current experiment, D50 treatments had greater pH, and yeast and mold counts compared with D100 silage. The lower production of total acids may have limited pH decline in D50 silages. Additionally, the lower lactic acid concentration in poorly packed silage observed in the current study was likely related to delayed growth of lactic acid–producing bacteria due to greater oxygen entrapped in the silage mass (Pahlow et al., 2003), allowing for greater yeast and mold proliferation in comparison to D100. In general, D100 and D75 had a more extensive fermentation than D50, evidenced by lower pH and greater production of organic acids. Greater packing in silage removes oxygen and impairs air penetration in the forage mass (Borreani et al., 2018), encouraging a desirable fermentation, promoting lactic acid bacteria growth, increasing organic acid production, and promoting a rapid decrease in pH, which can delay yeast and mold growth (Pahlow et al., 2003).

Effects of chemical additive and packing density on the nutrient composition and dry matter recovery of ensiled cocktail forage mix are shown in Table 3. Although there were greater yeast and mold counts in D50 (Table 2), silage had minimal changes in nutrient composition regardless of treatment. Silage treated with ADD had greater (*P* = 0.01) DM concentrations compared with CON, whereas DM recovery was lower (*P* = 0.04) for ADD than CON. Despite the effects of DM recovery for ADD-treated silages, all cocktail mix silages treatments had DM recovery values above 99% DM and this effect is unlikely to be biologically meaningful.

**Table 3:** Effects of chemical additive and packing density on the nutrient composition and DM recovery of ensiled cocktail forage mix^1^

Item^2^	CON			ADD			SEM^3^	*P*-value		
	Full	75%	50%	Full	75%	50%		AD	DEN	AD × DEN
DM, % as fed	39.9	40.0	39.2	41.7	42.0	41.3	0.92	0.002	0.49	0.99
DM recovery, % of DM	99.2	99.3	99.3	98.8	99.2	99.2	0.19	0.04	0.13	0.43
Ash, % of DM	9.7	9.4	9.5	9.5	9.5	9.9	0.19	0.29	0.18	0.16
WSC, % of DM	5.0	5.0	4.8	4.8	5.2	5.3	0.20	0.30	0.38	0.06
EE, % of DM	4.4	4.2	4.2	4.3	4.2	4.2	0.09	0.33	0.31	0.66
CP, % of DM	12.7	12.6	12.7	12.5	12.6	12.5	0.24	0.52	0.97	0.82
aNDFom, % of DM	49.8	50.6	50.6	50.8	50.5	50.2	0.76	0.70	0.90	0.51
Starch, % of DM	2.8	2.7	3.3	2.8	2.5	3.0	0.27	0.31	0.06	0.82
NDFD_30h_, % of aNDFom	69.4	69.6	68.5	67.9	68.7	68.3	0.70	0.06	0.31	0.49

1 Treatments consisted of AD: effect of additive (CON: 30 mL of distilled water; ADD: sodium sulfite, sodium metabisulfite, and fungal amylase; 0.11% of fresh forage); DEN: effect of packing density [D100 (maximum amount of material in laboratory silo), D75 (75% of full density, as-fed basis), and D50 (50% of full density, as-fed basis)].

2 WSC = water-soluble carbohydrates; EE = ether extract; aNDFom = neutral detergent fiber treated with α-amylase and sodium sulfite, corrected for residual ash; NDFD_30h_ = neutral detergent fiber digestibility after 30 h of incubation in rumen fluid.

3 Greatest SEM.

Overall, this study highlights the importance of well-packed silage. Even though fermentation was adequate for all silage samples, poorly packed cocktail forage mix silages had greater yeast and mold counts and a less pronounced fermentation. Contrary to our hypothesis, the use of a chemical additive restricted bacterial fermentation and did not improve aerobic stability. Future studies must evaluate the efficacy of other chemical and bacterial additives as fermentation modulators in cocktail mix silage.
